# Social media and control of soil-transmitted helminthiasis in Bhutan

**DOI:** 10.1371/journal.pntd.0007102

**Published:** 2019-02-28

**Authors:** Sangay Thinley, Kencho Namgyal, Antonio Montresor

**Affiliations:** 1 Programme Manager, Comprehensive School Health Programme/STH Programme, Department of Public Health, Ministry of Health, Thimphu, Bhutan; 2 WASH Specialist, UNICEF Country Office, Pyongyang, Democratic People’s Republic of Korea (formerly WASH officer with UNICEF Country Office, Bhutan); 3 Department of Control of Neglected Tropical Diseases, World Health Organization, Geneva, Switzerland; IRNASA, CSIC, SPAIN

Social media are interactive computer-mediated technologies that facilitate the creation and sharing of information, ideas, interests, and other forms of expression via virtual communities and networks [1]. They are also an important platform from which to promote health issues, for example, to facilitate the exchange of information and experiences among medical personnel and patients or among patients themselves, and to share health education messages. This opportunity is largely used by medical personnel, especially those in countries with good access to the internet [2].

We summarize here an experience in Bhutan, the country that introduced a school deworming programme in 1988, but records about frequency, drug administration, and coverage of the intervention are not available for the first 10 years; since 1998, a dose of albendazole was administered every six months to students through the school-based approach and preschool children are dewormed during their visit to health center; coverage is estimated between 80% and 95%. In 2004, a survey was conducted and the Soil Transmitted Helminth (STH) prevalence was evaluated at 24% [3].

In January 2012, Mr. Kencho Namgyal, former WASH Officer with UNICEF Bhutan Country Office, initiated the Facebook group “Health Nutrition and WinS (WASH in Schools)–Bhutan” [4] as an informal platform to exchange knowledge among personnel involved in school health activities, such as school health coordinators, teachers, school principals, administrators, civil servants, employees of nongovernmental organizations, international intergovernmental organizations, and United Nations agencies.

This Facebook group focuses on different aspects of school health, hygiene, and sanitation, including safe use of toilets, access to safe drinking-water, handwashing practices, personal hygiene (including menstrual hygiene), disease prevention (including periodical deworming, Weekly Iron Folic acid supplementation, Vitamin A supplementation, and oral health), waste management in schools, and food hygiene.

The intent of the group was to allow the sharing of ideas about best practices, challenges, and innovation at school and national levels and to ensure that children practice key hygiene behavior. The group has been highly successful: presently, more than 3,000 members are part of the group and, for example, in September 2018 alone, 50 posts were uploaded, mainly by teachers, containing more than 320 photos and videos showing WASH and health interventions in schools (frequently showing deworming campaigns), and in occasion of the 2018 Global Handwashing Day (15 October), over 60 posts were uploaded by different members of the group that received in total over 1,000 ‘likes’.

The platform managers, at least weekly, monitor the activity of the group and upload posts announcing training opportunities or health-related events (World Health Day, World Toilet Day, Global Menstrual Hygiene Day, among others) to ensure wide distribution of the information among teachers and active participation by schools.

Sharing of this locally driven, innovative solution has fostered horizontal exchange of knowledge and inspired schools in overcoming several barriers, such as limited resources and poor access to the services.

The members of the group commonly access Facebook through mobile phones that are widely used in Bhutan. The group also facilitates peer-to-peer contact whereby experienced personnel respond directly to requests from less experienced colleagues and quickly provide suggestions on how to solve practical problems. Such peer-to-peer exchange is yielding very efficient support for teachers in need and facilitating the work of the managers of school health programmes who would not otherwise have the possibility to promptly respond to individual requests for clarification from teachers.

In addition, information about similar activities conducted in other areas of the country is stimulating healthy competition among schools and reducing the sense of isolation frequently perceived by teachers in remote areas.

A recent STH survey conducted by the Ministry of Health (MoH) showed a dramatic reduction in STH prevalence in all areas of the country [5]; it is, however, difficult to distinguish the relative contribution of the different components of the programme (preventive chemotherapy, improvements of sanitation standards, and education for behavioral changes).

We consider this experience in Bhutan to be extremely positive. Social media are easily accessible, widely utilized, available at extremely low cost, and easy to realize. Furthermore, they could be easily implemented in other countries to facilitate preventive chemotherapy campaigns, not only for hygiene promotion and control of soil-transmitted helminthiasis alone, but also against other neglected tropical diseases.

Further research could be conducted among the participants to the Facebook group to evaluate in detail the benefits of the approach in terms of motivation and inspiration for teachers and students.

**Box. Advantages and disadvantages of social media use for promoting school health activities**

**Advantages**

1. In NTD control and Health and Hygiene promotion programmes, the use of social media can provide an efficient platform to reinforce social change through exchange of information in all directions (from management to implementers, from implementers to management, and among implementers and peers).
2. Social networks can be established with minimal cost.
3. The use of social media is informal and direct; implementers on NTD control programmes have normally much less reluctance in making requests or in expressing their concerns and criticisms than in a formal correspondence with hierarchical superiors.
4. Use of social media also promotes social accountability for users to demand services and for the national government to regulate, plan, and provide financial resources and for the local government to deliver services, such as basic water and sanitation services and NTD control programmes based on national standards.

**Disadvantages**

1. Participants should have easy access to the internet to benefit from the tool; the internet coverage is not always available in all the parts of the countries endemic for NTD, and not all of the potential participants have a personal smartphone. This could result in exclusion of the more remote and underserved areas and of the more unprivileged implementers.
2. Financial cost for starting social network are minimal but the virtual group with several hundred contributors should be managed almost daily to remain efficient and useful, and this can require a significant amount of time by the person responsible.
